# Book Reviews

**Published:** 1807-09

**Authors:** 


### CRITICAL ANALYSIS

OF THE

### RECENT PUBLICATIONS

ON THE

### DIFFERENT BRANCHES OF PHYSIC, SURGERY, AND MEDICAL PHILOSOPHY.

*Medical Reports of Cases and Experiments, with Observations, chiefly derived from Hospital Practice. To which are added, An Inquiry into the Origin of Canine Madness; and Thoughts on a Plan for its Extirpation from the British Isles.* By SAMUEL ARGENT BARDSLEY, M. D. M. R. S. Edin. and M. S. London, Physician to the Manchester Infirmary, &c.

NOTHING can be more desirable than the result of inquiries instituted with judgment, conducted with accuracy, and related with fidelity. If the Author is possessed of genius sufficient so to generalize the result of his facts, as to form practical conclusions, his works must for ever remain as so much added to that general stock of knowledge, which every student or writer may turn to his own use. Such appears to us the value of Dr. Bardsley's labours. It is not our intention to compliment the Author on any fuperior genius, the nature of the work before us would not admit of its display. It is sufficient, that all which was promised is accomplished; and without further preface, we shall proceed to show how much advantage the science may receive by such well-digested Reports.

Chronic rheumatism, the dreary companion of these humid regions, particularly in the north, and among sedentary manufacturers, is the first complaint of which our Author treats. The frequency of its occurrence has, it seems, induced the managers of the Infirmary, in compliance with the wishes of the medical gentlemen, to furnish the house with every means of curing or alleviating the sufferings of the patients. Warm and vapour baths, and well-constructed Galvanic and electrical apparatus, are always at hand, and have been judiciously applied.

"It would," says our author, "be trifling and useless to enter into any detail of the generality of the cases which have been submitted to my care. Indeed, the history of chronic rheumatism and its treatment, are both too well known to afford an expectation of the discovery of much novelty either of principles or facts; but still I am of opinion, that the communication of the general result of the treatment (under circumstances favorable both for practice and accurate observation) of a large number of cases, will be likely to prove useful, by establishing on a firmer basis, some of the various modes which have been generally recommended ed for the cure of this disease. It will be proper to premise, that I mean to include under the term chronic rheumatism, such painful affections of the muscular fibres, membranes, and joints, as are unattended with fever, specific virus, or peculiar derangement of the stomach and bowels; and which are seldom accompanied with external tumour or inflammation, but are very liable to shift suddenly from one part to another, and are readily propagated along the course of the membranes and muscular fibres. This definition will include chronic lumbago, sciatica, and what has been considered a distinct disease,—nodosity of the joints.

"No doubt the violence and inveteracy of chronic rheumatism are modified by the peculiar structure of the part which it affects. When the large joints with their ligaments, such as the hip joint and lumbar vertebræ become affected, the contiguous nerves often suffer from the same cause, and a long train of aggravated symptoms arise, which are with great difficulty subdued.

"The practice therefore must be regulated according to these varying circumstances; but I think myself entitled to assert, from a large induction of facts, that the rationale of the practice in the cure of every species of chronic rheumatism is, in general, simple and uniform. It consists in removing passive inflammation, and restoring the debilitated vessels and muscular fibres to their due tone and action. These ends are chiefly to be accomplished by topical applications, although internal remedies are by no means to be neglected.

"I find from an inspection of my medical case book, that two hundred and sixty-nine persons, (exclusively of a considerable number which were of a mixed and doubtful nature) labouring under severe chronic rheumatism, have been admitted under my care as in-patients of the Infirmary.

"The majority of these cases were of long standing, and had been brought from distant parts of the country to the Infirmary, as the last resource, after the usual means had failed."

In considering the comparative value of hot, tepid, or vapour bathing, the preference is decidedly given to the last, applied topically or generally. The mode of application is very accurately described, and does great justice to the liberality of the institution and attention of the practitioners. Electricity, as in most faithful reports, was found a very uncertain though sometimes a most powerful remedy. Galvanism, as might be expected, stands on similar ground. From topical bleeding and issues, very essential benefits were derived, insomuch that the Author particularly recommends both, as the first step in young and tolerably healthy subjects;—even in obstinate sciatica, Doctor Bardsley found great advantages from issues. His plan sometimes was almost to surround the joint with these drains, and as the discharge and irritation were insufficient, or too great, to increase or dry them up. By this judicious and close attention, he found many very obstinate cases yield to these well conducted remedies; blisters blisters and rubifacients were found highly serviceable in those pains which affected only the fascia. Under this head two very useful *formulæ* are given, the one of a stimulating epithema, the other of a liniment; the latter was found preferable, and very much to aid the use of vapour in cases of lumbago and sciatica.

The patient will perhaps hear, with less disappointment than the practitioner, that our author found so little effect from internal remedies. He divides them into sudorifics and stimulants. From neither of these was sufficient advantage derived to encourage a persevering use of them. But there are a few other remedies, whose *modus operandi*, to use the author's expression, is not so easily ascertained, and the advantages of which we cannot help wishing, had made a more considerable object in these inquiries. We refer to Peruvian bark and arsenic. That there is some correspondence in the actions excited by these remedies, however different they may seem in most of their known properties, is very certain by the effects each of them produces on intermittent fevers. The testimonies in their favour, in the various stages of rheumatism, are so powerful, that we are utterly at a loss to account for the few experiments we meet with, in a work so professedly designed to try the effects of various remedies. This is particularly to be regretted, because in the subsequent table, we have, doubtless, a very faithful report of the cases, the remedies, and, as it would seem, the comparative value of the latter. But if more cases have been submitted to one class of remedies than to another, the inference is obvious that we may see more cures under those articles, though the comparative value may be less. Bark indeed seems to have had more opportunities of fair trial; but after the recommendation of arsenic from a quarter so highly respected* by the Author, we were prepared to expect a more frequent trial of that important remedy, especially as some success was experienced in the few cases in which it was used. The following are the Author's concluding remarks on this subject.

" I am therefore disposed to infer (as far as my limited experience will permit) that it is only in the protracted chronic rheumatism, where the vital powers are much diminished, and the ends of the bones, periosteum, capsules or ligaments of the joints, are likewise partially affected, that the use of arsenic is likely to prove eminently successful. However, there can be no doubt, from its indisputable efficacy in such instances as have already been published, of the propriety of instituting a number of comparative trials of this remedy, with others of an approved and established kind, in the cure of every variety of so prevalent and disstressing a malady as chronic rheumatism. It is my intention to enter, at no distant period, upon this experimental inquiry. I feel more encouraged to this undertaking, from a conviction, the

---

* See Mr. Jenkinson's papers on this subject in our Journal.

result of frequent and attentive observation, that arsenic is a safe and harmless remedy, when *prudently* administered."

From turpentine less relief was derived than some writers have promised, but the cod-liver-oil was sometimes useful. A single case of nodosity of the joints, as described by Doctor Haygarth, was with much assiduity at last subdued by salivation, topical bleeding, and restorative remedies.

Two cases follow, of a *singular affection of the calves of the legs*, which we shall transcribe, as we do not remember to have met with such in our reading or practice.

"John Lindsay, weaver, aged 36, was admitted an In-patient of the Infirmary, April 3, 1796, with the following complaint. He was seized three weeks ago with a violent pain in the muscles of the calf of each leg, soon after his removal from a garret into a damp cellar, where he worked. The pain was almost incessant; it was not increased by pressure; but became so severe at night, as to deprive him of sleep. The disorder was entirely confined to the parts first attacked; and was unattended with discoloration, tumor, or any external appearance of disease, except that the muscles were unusually flaccid and shrunk. He was able to walk about the wards, and conceived this exercise afforded him a temporary respite from pain. The appetite was tolerably good, and the animal functions in general but little impaired. Pulse ninety-six, and rather feeble. His countenance exhibited symptoms of uncommon anxiety aed distress."

This patient died suddenly the third day after admission.

"James Mansel, joiner, aged 30, admitted an In-patient, February 6th, 1799. He first experienced, six weeks since, a soreness in the calf of each leg, which gradually increased, so as to prevent him following his employment. The pain was most severe in the night, but never entirely quitted him. He frequently has sat up the whole night in bed, endeavouring by pressure, and handling the muscles, to relieve his pain. He has not been affected with chronic rheumatism, and is totally unable to account for the origin of this disease. The pulse and most of the natural functions are regular; but the patient's countenance bespeaks great distress, and he is much reduced by pain and want of sleep. As I could have no doubt of the similarity of the present to the preceding unfortunate case, I entered upon its treatment with great caution. The parts were enveloped with flannel, and fomented with hot water. Leeches were also applied, and diaphoretics with opium prescribed internally. This plan was attended with no benefit. The pain increased to a distressing degree in the night, and seemed indeed, to be aggravated by opiates. Blisters were then ordered to each calf of the leg; and when the vesicated parts healed, the blisters were renewed. At the same time electrical sparks were drawn from the neighbourhood of the affected muscles; and slight shocks passed through them. Bark and Guaiacum were also exhibited. By this method the pains gradually subsided; and in three weeks, the patient was discharged entirely cured."

A case of rheumatism from taking cold under the use of mercury, was cured by pushing that remedy to its utmost extent.—We sincerely wish, under this head of chronic rheumatism from mercury, that Dr. Bardsley had made two divisions. One is, where a patient is suddenly seized with cold during the use of that remedy; the other, where pains in the joints follow the repeated use of the remedy, without any improper exposure. The first may very probably be cured in the manner proposed by our Author, but we have always seen the latter exasperated in the end, though for a time somewhat relieved, by every repetition of mercury in any form.

The next subject considered is Diabetes Mellitus. On this we meet a number of truly valuable remarks, and eight cases related with an accuracy which will only weary those who are undeserving the labour bestowed on them by the Author. A variety of incidents show the advantage of confining the patient to animal food; but after all the diligence of Dr. Bardsley, we confess ourselves at a loss to determine whether the few cures that are established, are to be imputed to any of those remedies, or to those spontaneous changes which so often occur in chronic diseases. We are not, however, less thankful for the minute accuracy of the reports, and shall certainly avail ourselves of them as often as these dreary cases occur. We ought to mention, that some useful experiments are added to our chemical knowledge of diabetic urine.

The effects of Galvanism in paralysis are next mentioned. Of these, our limits will not admit us to give more than the Author's conclusions.

"It appears," says Dr. B. "sufficiently evident from the above recital of facts, that the Galvanic stimulus is an efficacious, though not certain remedy in paralytic affections. I have long been in the practice of employing Electricity in the treatment of Paralysis, and sometimes with the happiest result. But as far as my hitherto limited experience will permit me to institute a comparison between the effects of Galvanism and Electricity in Paralysis, I am inclined to prefer the employment of the former to the latter, in all cases which appear to originate solely from a diminished state of excitement in the sensorium. I have repeatedly had recourse to Electricity for the relief of such symptoms as are described in Mc Cartney's case, where the long continuance of the loss of speech, distortion of the muscles of the face, and depravation of the intellectual faculties, denoted a high degree of collapse, or impaired energy of the brain; but never with such speedy and decisive advantage as was obtained from the application of Galvanism. Indeed the superior efficacy of the Galvanic to the Electrical influence, was in Mc Cartney's case fairly put to the test. No perceptible advantage was derived from an assiduous application of electricity, for more than a week previous vious to the exhibition of Galvanism. It is certainly extremely difficult to point out any characteristic signs by which we may be enabled to discriminate between those affections of the brain, which depend merely upon diminished excitement, and those which originate from organic derangement of an incurable kind. Wherever Paralysis arises from tumors compressing the substance of the brain, or from a diseased alteration in its mass and structure, or from exstravastion of a fluid in such a state or degree, as will not admit of its absorption; it will be readily admitted, that no benefit is to be expected from the employment of Galvanism, or perhaps any other remedy yet discovered. But what are the diagnostic signs of such incurable conditions of the brain? I confess myself unable to point them out with any certainty. I may venture to state, however, from a few observations on this subject, that such affections of the brain are for the most part insiduous in their approach, gradual in their progress, and when once formed, they admit of little, if any, exacerbation or remission in their symptoms. But although no benefit can be rationally expected to accrue from Galvanism in such a calamitous state of the brain as has been just described; I am not aware that a *cautious* and *prudent* use of the remedy will prove certainly injurious. I am, however, enabled to affirm, that in those cases of Paralysis, where sense, motion, and intelligence, are greatly impaired, it is always imprudent to push the experiment beyond certain limits. If the patient's rest, appetite, and feeble sense of enjoyment, be evidently disturbed; if tremors, convulsive sobs, and tears, with other signs of increased irritability, should either immediately, or soon after the operation, supervene, it will be proper at least to suspend, if not altogether discontinue, the use of Galvanism. The necessity for caution is exemplified in the following brief statement of a fact which lately occurred. A young lady (the daughter of a late eminent physician) had laboured under a general Paralytic affliction (which most probably originated from an attack of Hydrocephalus internus, when an infant) for many years; and which had progressively deprived her of all muscular exertion and the power of speaking louder than a whisper. She had some years before, upon her father's consulting me on the subject, submitted to a seven weeks trial of electricity, under every form and degree which I considered most likely to be beneficial. No advantage was derived from the experiment. It was then my opinion, that the complaint depended on some internal organic derangement of the sensorium. She was, however, strongly recommended last year, when at Bath, to make a trial of Galvanism. The experiment, I have every reason to believe, was skilfully conducted. Its effects, however, were not only unproductive of any advantage, but very distressing to the patient's feelings, and injurious, for a time, to her general state of health.

"It may not be improper to remark here, by way of caution, that that I have been more than once disappointed in communicating the Galvanic influence to a patient, notwithstanding the skin had been duly prepared by moistening it with a proper mixture of nitric acid and water, and covering it with metallic leaf, and although other patients, treated in a similar manner, had experienced at the same time, the Galvanic sensation in its due force. This failure I found to arise from some peculiar condition of the skin (perhaps increased thicknees?) for upon the application of the wire conductors to another part of the same limb, which had been in like manner prepared, as in the first attempt, I was able to succeed with the greatest ease.

" *Result of the Effects of Galvanism in Paralysis, &c.*

" Five Cases of general Paralysis, of which,

| | |
|---|---|
| Cured - - - - - - | 3 |
| Relieved - - - - | 1 |
| Not Relieved - - - | 1 |

" *Five Cases of Partial Paralysis.*

| | | |
|---|---|---|
| Ischuria - - - | Cured - - - | 1 |
| Hemiplegia - - | Cuied - - - | 1 |
| Hemiplegia - - | Not Relieved - | 1 |
| Paraplegia - - | Cured - - - | 1 |
| Paraplegia - - | Much Relieved | 1 |

Hip Joint Case, One Cured."

Dr. B. next considers the effects of oxyd of bismuth in pyrosis and other diseases of the stomach. Dr. Marcett's observations are amply confirmed. It is further remarked, that valuable as this remedy is found in the above mentioned complaints, it is less to be depended upon in the general class of *neuroses* than the other metallic oxyds.

The Work concludes with some valuable remarks on Rabies, and the means of exterminating this dreadful malady from the British isles. Whether this is practicable we pretend not to determine, but are ready to give every credit to our Author for his good intentions, and to entreat that he may meet with that assistance from us all, to which his labours entitle him, and which the importance of the subject demands.

---

*Practical Observations on the Uterine Hæmorrhage: with Remarks on the Management of the Placenta.* By John Burns, Lecturer on Midwifery, and Member of the Faculty of Physicians and Surgeons in Glasgow.

It is somewhat surprising we should have been so long without any tract devoted exclusively to this important subject. Mr. Burns begins with a short description of the connection between the uterus and ovum, and the causes of hæmorrhage during gestation; the manner in which Nature endeavours to relieve them; the difficulculty which attends that attempt; and the necessary consequence of frequent returns of the complaint.

The author next enters, with more precision, into the causes of uterine hæmorrhage under gestation. These he considers, as imputable to external violence, producing a separation of part of the ovum; to fatigue, or over exertion; which, by their effects on the parts, or on the general circulation, may produce such an effect; straining the abdominal muscles from any cause; a preternatural degree of action in the vessels going to the placenta, or decidua arising from general plethora, or some peculiarity in the state of the parts; a want of correspondence between the action of the uterus and ovum; spasmodic action of the os uteri; any cause which may interrupt the progress of gestation, or the formation of that jelly which ought to be secreted before the os uteri — in some cases, the author has observed a change in the structure of the placenta near the separated part; lastly, the insertion of the placenta over the os uteri. This last he considers with other practitioners, as the most common of all the causes producing uterine hæmorrhage during gestation.

The next object attended to, is the *effect* of Uterine Hæmorrhage, which leads to a consideration of the prognosis.

As the reputation of the practitioner depends often more on this last, than even on his success, we shall transcribe this part of the work, as a specimen of the Author's style.

"We may lay it down as a general observation, that few cases of profuse hæmorrhage, occurring in an advanced stage of gestation, can be cured without delivery or the expulsion of the child. For when the discharge is copious or obstinate, the placenta is generally separated, sometimes to a very considerable extent, and a re-union, without which the woman can never be secure against another attack, can rarely be expected. If the placenta present, the hæmorrhage, although suspended, will yet to a certainty return, and few will survive if the child be not delivered.

"But in those cases where only a portion of the decidua, or a little bit of the margin of the placenta, has been detached, and the communicating vessels opened, either by a state of over action in the vascular system, or by too much blood in the vessels, or by some mechanical exertion; then, if proper care be taken, the hemorrhage may be completely and permanently checked; or if it should return, it may be kept so much under, or may consist so much of watery discharge from the glands about the os uteri, as neither to interfere with gestation, nor injure the constitution; yet it is to be recollected, that even these cases of flooding may sometimes proceed to a dangerous degree, requiring very active and decided means to be used; and in no case can the patient be considered as safe, unless the utmost care and attention be paid to her conduct.

"It would thus appear, that some hemorrhages almost inevitably end either in the delivery of the child, or the death of the parent; parent; whilst others may be checked or moderated without an operation. A precise diagnostic line, liable to no exceptions, cannot be drawn betwixt those cases; and, therefore, whilst we believe that rapid and profuse hemorrhagies, which indicate the rupture of large vessels, can seldom be permanently checked, we still, provided the placenta do not present, are not altogether without hopes of that termination which is more desirable for the mother, and safer for the child, than premature delivery. In slighter cases, our hope is joined with some degree of confidence.

" A second attack, especially if it follow soon after the first, and from a slight cause, greatly diminishes the hope of carrying the woman to a happy conclusion without manual interference.

" In forming our opinion respecting the immediate danger of the patient, we must consider her habit of body, and the previous state of her constitution. We must attend to the state of the pulse, connecting that in our mind with the quantity and rapidity of the discharge.

" A feeble pulse, with a hæmorrhage, moderate in regard to quantity and velocity, will, if the patient have been previously in good health, generally be found to depend on some cause, the continuance of which is only temporary.

" But when the weakness of the pulse proceeds from profuse or repeated hæmorrhage, then, although it may sometimes be rendered still more feeble by oppression, or feeling of sinking at the stomach; yet, when this is relieved, it does not become firm. It is easily compressed, and easily stopped by motion; or, sometimes, even by raising the head.

" If the paroxysm is to prove fatal, the debility increases—the pulse flutters—the whole body becomes cold and clammy—the breathing is performed with a sigh—and syncope closes the scene.

" If irritation be conjoined with hæmorrhage, then the pulse is sharper, and, although death be near, it is felt more distinctly than when irritation is absent.

" The termination in this case is often more sudden than a person, unacquainted with the effect of pain or irritation on the pulse, would suppose. For when the pulsation is distinct, and even apparently somewhat firm, a slight increase of the discharge, or sometimes an exertion without discharge, speedily stops it, the heat departs, and the patient never gets the better of the attack.

" We must likewise remember, that a discharge, which takes place gradually, can be better sustained than a smaller quantity, which flows more rapidly. For the vessels in the former case come to be accustomed to the change, and are able more easily to accommodate themselves to the decreased quantity. But when blood is lost rapidly, then very speedy and universal contraction is required in the vascular system, in order that it may adjust itself to its contents, and this is always a debilitating process. The difference too betwixt the former and the present condition of the body, is rapidly produced, and has the same bad effect as if we

 were were instantly to put a free liver upon a very low and abstemious diet.

"In all cases of flooding, we find that during the paroxysm, the pulse flags, and the person becomes faint. Complete syncope may even take place, but this in many cases is more dependent on sickness or oppression at the stomach, than on direct loss of blood. In delicate and irritable habits, the number of fainting fits may be great, but unless the patient be much exhausted, we generally find that the pulse returns, and the strength recruits. The prognosis here must depend greatly on the quantity and velocity of the discharge; for it may happen, that the first attack of hæmorrhage may produce a syncope, from which the patient is never to recover."

Having thus explained the causes, effects, general symptoms, and probable event of Uterine Hæmorrhage, Mr. Burns enters on the Treatment. In this, he is as minute as the nature of the subject requires, but not more diffuse. To enumerate therefore, all contingencies which the student is taught to expect, and all the remedies recommended under each, would be to transcribe the whole. Such means of relief, as depend on manual assistance, are reserved for the succeeding chapter, "On Delivery." This comprehends all the circumstances under which Delivery should be prematurely attempted; after which, the author describes the effects of the presentation of the placenta at the period of parturition, and the manner in which the practitioner should conduct himself under such an event.

A chapter follows of Hæmorrhage during Labour, and another on the same event after Delivery, and after the expulsion of the Placenta; and lastly, on the Management of the Placenta. These concluding chapters necessarily embrace every part of midwifery which is any way connected with the subject. All the directions given by the author are blended with those cautions, which cannot be too often impressed on young practitioners, and with that decision, which the nature of the case will always demand.

This work, though containing but little novelty, may be considered as a very valuable practical epitome of all that is required of the practitioner, for the relief of uterine hæmorrhage during gestation or parturition.

